# Hypoglossal Nerve Stimulation in the Context of Non-CPAP Therapy in Patients With OSA: Improvement in the Apnea-Hypopnea Index in Randomized Controlled Trials

**DOI:** 10.1016/j.chest.2025.09.132

**Published:** 2025-10-14

**Authors:** Mohammed Azib Zahid, Deeban Ratneswaran, Joerg Steier

**Affiliations:** a Southend University Hospital, Mid and South Essex NHS Trust, Westcliff-on-Sea, Southend-on-Sea, Essex, England; b Faculty of Life Sciences & Medicine, King’s College London, London, England; c Lane Fox Unit/Sleep Disorders Centre, Guy’s & St Thomas’ NHS Foundation Trust, London, England

OSA affects up to 1 billion people worldwide, with about 40% of patients presenting with moderate to severe disease.^1^ CPAP therapy is the treatment of choice; it provides positive pressure to the airway through an interface, preventing upper airway collapse in the asleep patient. However, CPAP adherence varies substantially, between 46% and 83%.^2^

In addition to other non-CPAP therapies, hypoglossal nerve stimulation (HNS) has emerged as a novel approach for treating OSA. The rationale of this communication was to review the current evidence base of well-designed randomized clinical trials using electrical stimulation to treat patients with OSA.

## Methods

MEDLINE/PubMed and the Cochrane Central Register of Controlled Trials databases were searched to identify randomized controlled trials (RCTs) investigating electrical stimulation of the upper airway in patients with OSA. Trials with a primary outcome focusing on upper airway patency (apnea-hypopnea index [AHI]/oxygen desaturation index [ODI]) up to October 2024 were included.

## Results

The included studies varied considerably in design. The Stimulation Therapy for Apnea Reduction (STAR) trial, which followed an enhanced enrollment randomized withdrawal design, used drug-induced sleep endoscopy (DISE) to prescreen patients who were likely to respond.^3^ Studies using the noninvasive method focused on unselected cohorts of patients with OSA; post hoc responder analyses were then performed. Overall, 46 participants in the STAR trial underwent the randomized therapy withdrawal phase.^3^ The more recently targeted hypoglossal nerve, in the Targeted Hypoglossal Nerve Stimulation 3 (THN3) trial,^4^ used a multicenter, randomized design with all patients undergoing implantation and assigned 2:1 to early (month 1) or delayed (month 4) activation. This approach allowed group comparisons at month 4 and a cohort analysis following 11 months of completed therapy (at month 12 or 15, respectively).

Another study, the Effect of Upper Airway Stimulation in Patients with Obstructive Sleep Apnea (EFFECT) trial, adopted a double-masked, sham-controlled, crossover design with analysis after 1 and 2 weeks, showing improvements in AHI, oxygenation metrics, and patient-reported outcomes.^5^ Finally, the Australian Clinical Study of the Apnex Medical HGNS System to Treat Obstructive Sleep Apnea (Apnex) clinical trial was designed to evaluate the Apnex Medical HNS system (unilateral, invasive); it was terminated early, however, and did not formally report any outcomes data.^6^

The Transcutaneous Electrical Stimulation for OSA (TESLA) trial used a randomized crossover study design assessing 1 night of transcutaneous treatment vs a sham night with patients acting as their own control.^7^ The TESLA home trial^8^ described 3-month outcomes compared with usual care (ongoing CPAP in patients with low adherence). Wu et al^9^ conducted a crossover RCT that compared intermittent and continuous transcutaneous electrical stimulation (Table 1).^3^, ^4^, ^5^, ^6^, ^7^, ^8^, ^9^

**Table 1:** Randomized Controlled Trials Evaluating HNS in OSA and the Effect on the AHI

Study (Author, Year)	Randomized Patients (n)	Age (y)	Sex (% Male)	Baseline AHI (Events/h)	Stimulation Type	Eligibility Criteria	AHI Reduction (Events/h) [95% CI]	Targeted Muscle	Adverse Effects	Patient-Reported Outcome Measures	Study Outcome Measures
STAR (Strollo et al,^3^ 2014)	46	54.5 ± 10.2	83	30.7 ± 10.3	Unilateral invasive	Age: ≥ 22 y (adults) Sex: Both male and female BMI: Patients exceeding study-specified BMI limits were excluded (exact range not provided) Diagnosis: Clinical history and physical examination suggestive of moderate to severe OSA (with PSG confirmation implied)	–16.4 [–21.3 to –11.5]	Genioglossus, specific	Procedure-related SAEs, occurring in < 2% of participants Minor: Various transient events (eg, temporary discomfort and stimulation-related side effects)	ESS, FOSQ, EQ-5D, VAS	ODI decreased by approximately 70% (from 25.4 events/h to 7.4 events/h); reduced time with Spo_2_ < 90%
TESLA (Pengo et al,^7^ 2016)	36	51.9 ± 10.2	72	24.0 ± 10.7	Bilateral transcutaneous	Age: > 18 y to < 75 y Sex: Both sexes BMI: > 18 kg/m^2^ and < 40 kg/m^2^ (excluding BMI < 18 kg/m^2^ and > 40 kg/m^2^) Diagnosis: OSA defined by an ODI ≥ 15 events/h or ODI ≥ 5 events/h with symptoms (eg, ESS score > 10)	–9.0 [–16.2 to –1.8]	Genioglossus, unspecific	No SAEs were reported Minor: Mild, transient local effects such as minor skin irritation at electrode sites	SSS, VAS	ODI decreased from a median of approximately 26.9 events/h to 19.5 events/h (sham vs active). Minor improvement in overall oxygenation
EFFECT (Heiser et al,^5^ 2021)	89	57.5 ± 9.8	81	32.3 ± 11.4	Unilateral invasive	Age: ≥ 18 y (adults; no upper limit specified) Sex: Both sexes BMI: Not explicitly specified Diagnosis: Documented moderate to severe OSA based on PSG (baseline AHI approximately 32 events/h)	–15.5 [–18.3 to –12.8]	Genioglossus, specific	One stroke in the sham group (complete recovery); no other SAEs Minor: No additional side effects	ESS, FOSQ, PSS, CGI-I	ODI difference from baseline was –12.2 events/h (stimulation vs sham). Modest improved minimal O_2_ saturation and sleep time < Spo_2_ of 90%
Wu et al^9^ (2022)	15	47.2 ± 11.5	100	31.1 ± 12.3	Intermittent and continuous stimulation, transcutaneous	Age: Not explicitly defined (adult population; mean approximately 47 years) Sex: Male participants only BMI: Not specified Diagnosis: OSA or simple snoring diagnosed by clinical evaluation/PSG (variable severity)	–7.3 [–23.5 to –3.1] (continuous); –13.3 [–18.5 to 3.9] (intermittent)	Genioglossus, unspecific	No SAEs occurred Minor: transient discomfort at the stimulation site was observed	ESS, VAS	Median ODI reduction of 9.25 events/h with intermittent stimulation vs 3.3 events/h with continuous stimulation. Minimal changes in overall oxygen saturation
THN3 (Schwartz et al,^4^ 2023)	115	52.0 ± 11.0	80	35.9 ± 15.3	Unilateral invasive	Age: > 18 y Sex: Both sexes BMI: < 35 kg/m^2^ (patients with BMI ≥ 35 /m^2^ are excluded) Diagnosis: Moderate to severe OSA defined as AHI ≥ 20 events/h	–14.4 [–18.0 to –10.5]	Genioglossus, hyoglossus, styloglossus, specific	Two SAEs were reported, attributed to the implant procedure or study protocol Minor: A total of 100 minor events were recorded, reflecting expected procedural risks (eg, transient discomfort, minor local effects)	ESS, FOSQ, EQ-5D, VAS	At 4 mo, ODI responder rate (≥ 25% decrease) was 62.5% in the treatment group vs 41.3% in control participants. By 12-15 mo, ODI response was approximately 60% in the entire cohort
TESLA Home (Ratneswaran et al,^8^ 2023)	56	50.6 ± 9.8	76	24.5 ± 8.2	Bilateral transcutaneous	Age: 18-85 y Sex: Both sexes BMI: 18.5-32 kg/m^2^ Diagnosis: Mild to moderate OSA defined as AHI 5-35 events/h	–11.5 [–20.7 to –2.3] (unadjusted); –7.0 [–15.7 to 1.8] (adjusted)	Genioglossus, unspecific	No SAEs were observed Minor: One participant experienced a mild headache, with no other significant side effects	ESS, SSS, FOSQ	Reduction of ODI was –11.3 events/h (95% CI, –19.3 to –3.2; *P* = .007) unadjusted. When adjusted, the difference was –8.3 events/h (95% CI, –16.1 to –0.6; *P* = 0.036), favoring the intervention group
Apnex Clinical Study (2011-2013)^6^	132 planned	NA	NA	NA	Unilateral invasive (Apnex Medical HNS system)	Age: 21-80 y Sex: Both sexes (not explicitly restricted) BMI: ≤ 35 kg/m^2^ Diagnosis: Previously diagnosed moderate to severe OSA based on standard PSG criteria	NA	NA	Safety analysis (description of all adverse events at 12 mo) planned; no data reported due to early termination	Terminated in 2013; no posted data on outcomes	Planned to assess AHI and ODI changes at 6/12 mo; no results reported due to early study termination

Data are presented as mean ± SD. The effect size using invasive (STAR, THN3, and EFFECT) and noninvasive (TESLA, TESLA home, and Wu et al) approaches have significant overlap in the CIs. The terminated Apnex clinical study was designed to assess safety and efficacy but did not formally report outcomes data. The total number of studied patients using HNS in randomized controlled trials remains low. AHI = apnea-hypopnea index; CGI-I = Clinical Global Impression-Improvement; EFFECT = Effect of Upper Airway Stimulation in Patients with Obstructive Sleep Apnea; EQ-5D = EuroQol-5 Dimension; ESS = Epworth Sleepiness Scale; FOSQ = Functional Outcomes of Sleep Questionnaire; HNS = hypoglossal nerve stimulation; n = number of participants; NA = not applicable; ODI = oxygen desaturation index; PSG = polysomnography; PSS = Patient Satisfaction Survey; SAE = serious adverse event; Spo_2_ = arterial oxygen saturation by pulse oximetry; SSS = Stanford Sleepiness Scale; STAR = Stimulation Therapy for Apnea Reduction; TESLA = Transcutaneous Electrical Stimulation for OSA; THN3 = Targeted Hypoglossal Nerve Stimulation 3; VAS = visual analog scale.

## Discussion

Electrical stimulation is an emerging alternative treatment for patients with OSA with promising but limited data from RCTs. Other possible non-CPAP therapeutic options include weight reduction, positional therapy, and mandibular advancement devices. Furthermore, surgical options or maxillomandibular advancement surgery have been considered when anatomical factors significantly contribute to the airway collapse in patients with OSA.

### Invasive HNS

In the enhanced enrollment randomized withdrawal phase of the STAR trial (n = 46), deactivating HNS raised the AHI from 7.4 ± 6.1 events/h to 25.5 ± 15.2 events/h, whereas it remained at 8.9 ± 11.3 events/h with ongoing therapy.^3^ In the 5-year follow-up study, the median AHI fell from 33.2 events/h to 6.2 events/h (*P* < .001), and quality of life improved.^10^ However, the data presented analyzed a subset of the overall cohort (“responders”), and questions remain about the generalizability of the results for patients with moderate to severe OSA.

By comparison, the THN3 trial adopted broader entry criteria (BMI ≤ 35 kg/m^2^ and AHI 20-65 events/h) that did not require DISE for selection of potential responders.^4^ The STAR study (BMI ≤ 32 kg/m^2^ and AHI 20-50 events/h) excluded patients with concentric collapse of the upper airway.^3^ Similarly, the EFFECT trial, which enrolled 89 patients with moderate to severe OSA (AHI ≥ 15 events/h), reported an AHI reduction of 15.5 events/h (95% CI, –18.3 to –12.8) along with improvements in oxygenation metrics and patient-reported outcomes; follow-up duration was short (1-2 weeks of treatment), however.^5^

The Apnex clinical trial also deserves mention here.^6^ It was designed to evaluate the Apnex HNS system in patients who had not achieved or were intolerant of CPAP therapy but was terminated early with no formally reported outcomes data.

### Transcutaneous Approach

Reported follow-up periods of trials using the noninvasive method are limited. Pengo et al^7^ conducted a double-masked, sham-controlled crossover RCT (n = 36) using TESLA; a reduction was observed in the AHI of –9.0 events/h (95% CI, –16.2 to –1.8; *P* = .018) with 17 responders after 1 night in the sleep laboratory. Ratneswaran et al^8^ reported an unadjusted improvement in the AHI of –11.5 events/h (95% CI, –20.7 to –2.3) and in the Epworth Sleepiness Scale of –3.0 points (95% CI, –5.4 to –0.5) in 56 patients with mild to moderate OSA (BMI < 32 kg/m^2^) following 3 months of domiciliary treatment. Wu et al^9^ (n = 15) compared continuous vs intermittent stimulation protocols, yielding reductions of –7.3 events/h (95% CI, –18.5 to 3.9) and –13.3 events/h (95% CI, –23.5 to –3.1), respectively, following single nights of treatment.

Despite the available RCT data, data published from clinical follow-up studies regarding the transcutaneous approach are currently scarce. However, this approach may be used to “bridge” the time to implantation, during DISE to test “response,” and lends itself to a domiciliary trial prior to further selection of treatments.

### Health Economics

Pietzsch et al^11^ found that, despite substantial upfront costs, upper airway stimulation provides meaningful long-term quality-adjusted life year (QALY) gains, with an incremental cost-effectiveness ratio of approximately $39,471 per QALY in the US health care setting. The European analysis of HNS-related costs reported EUR 44,446 per QALY gained.^12^ Blissett et al^13^ reported that HNS lifetime benefits yielded an incremental cost-effectiveness ratio of around £17,989 per QALY gained in the United Kingdom.

### Other Studies in the Field

The Bilateral Hypoglossal Nerve Stimulation for Treatment of Obstructive Sleep Apnoea (BLAST OSA) trial evaluated bilateral HNS using the Genio system (Nyxoah) in 22 patients with OSA.^14^ At 6 months, investigators found a mean AHI reduction of –10.8 events/h (95% CI, –14.6 to –7.0) in patients with moderate to severe OSA who were CPAP intolerant; however, this trial lacked a control arm. The results of the Dual-Sided Hypoglossal Nerve Stimulation for the Treatment of Obstructive Sleep Apnea (DREAM) US trial using the Genio system, a single-arm interventional trial, reported that the co-primary endpoint was achieved by 77.4% (n = 113) at 12 months; findings included a reduction in the AHI of –18.3 events/h (95% CI, –20.8 to –15.8) and a serious adverse event rate of 8.7%.^15^^,^^16^

The eXciteOSA device (Signifier Medical Technologies) uses a different approach; it utilizes daytime intraoral neuromuscular electrical stimulation for 20 minutes daily for 6 weeks to target and train the genioglossus, geniohyoid, hyoglossus, and styloglossus muscles. Using this method, Baptista et al^17^ described a modest AHI reduction of –1.82 events/h (from 6.85 ± 4.44 events/h to 5.03 ± 3.82 events/h; *P* < .001) in 115 individuals with snoring and mild OSA.

### Other Non-CPAP Therapies

The European Respiratory Society guidelines on non-CPAP therapies highlighted the efficacy of custom-made dual-block mandibular advancement devices in reducing the AHI and improving oxygenation parameters in patients who are intolerant of CPAP, particularly in mild to moderate OSA.^18^ The guideline also assessed positional therapy showing a decrease in supine-dependent airway collapse, with reported improvements in the AHI. Furthermore, weight reduction strategies are an important long-term adjunct treatment for patients with OSA; a meta-analysis indicated that a 20% reduction in BMI was associated with an approximate 57% decrease in the AHI, alongside improvements in oxygen desaturation and sleep fragmentation.^19^ Of note, tirzepatide, a dual glucose-dependent insulinotropic polypeptide/glucagon-like peptide-1 receptor agonist, was tested in the Obstructive Sleep Apnea Master Protocol GPIF: A Study of Tirzepatide (LY3298176) in Participants With Obstructive Sleep Apnea (SURMOUNT-OSA) phase 3 trials with reported AHI reductions of –25.3 events/h (95% CI, –29.3 to –21.2) and –29.3 events/h (95% CI, –33.2 to –25.4) in 2 separate RCTs.^20^ Furthermore, an RCT on multilevel surgery reported a reduction in the AHI of –17.6 events/h (95% CI, –26.8 to –8.4) at 6 months.^21^

## Conclusions

The overall quality of the data for non-CPAP therapies from RCTs, according to the European Respiratory Society guidelines, remains low. There are sparse data from peer-reviewed RCTs to guide evidence-based therapeutic choices regarding the efficacy of HNS to improve the AHI.

## Financial/Nonfinancial Disclosures

The authors have reported to *CHEST* the following: J. S. is the named inventor for a patent (WO2016124739A1) for an apparatus for treatment of snoring and sleep apnea on behalf of his employer (Guy’s & St Thomas’ NHS Foundation Trust/King’s College London). J. S. has received National Institute for Health and Care Research funding for his research/group. None declared (M. A. Z., D. R.).
